# A dispute on antibiotic prophylaxis for surgical wound infections in clean and clean-contaminated surgery

**DOI:** 10.1097/MS9.0000000000002871

**Published:** 2025-01-07

**Authors:** Chao-Ming Hung, Ying-Yu Lee, Chong-Chi Chiu

**Affiliations:** a Department of General Surgery, E-Da Cancer Hospital, I-Shou University, Kaohsiung, Taiwan; b School of Medicine, College of Medicine, I-Shou University, Kaohsiung, Taiwan; c Department of Colorectal Surgery, E-Da Cancer Hospital, I-Shou University, Kaohsiung, Taiwan; d Department of Medical Education and Research, E-Da Cancer Hospital, I-Shou University, Kaohsiung, Taiwan

*Dear Editor,*

The issue of antibiotic prophylaxis in clean and clean-contaminated surgery has been a point of debate for a long time. This multifaceted discussion reflects various perspectives within the medical community. Tang *et al*’s updated systematic review and meta-analysis provide compelling evidence supporting the efficacy of prophylactic antibiotics in reducing surgical site infections (SSIs)^[1]^. However, it also raises critical questions about antibiotic stewardship, the necessity of such interventions, and the implications for clinical practice.

Advocates emphasize that reducing hospitalization length by nearly one day is more than just a mere statistic; it carries significant real-world implications for patient quality of life and healthcare efficiency. This seemingly slight decrease in hospital stay can substantially improve patients’ overall well-being, allowing them to return to their daily lives sooner and reducing the emotional and financial stress associated with extended hospitalizations. Furthermore, hospitals can optimize their resource allocation by minimizing the risk of SSIs through appropriate antibiotic use. This proactive approach not only enhances patient safety but also alleviates some of the pressures on healthcare systems that are increasingly strained by rising patient numbers. By ensuring that patients recover more quickly and reducing the incidence of complications, healthcare providers can focus their efforts on delivering high-quality care to more individuals. Ultimately, this strategy fosters a more sustainable healthcare environment, benefiting patients and providers.

Conversely, there is growing concern regarding the role of prophylactic antibiotics in contributing to antibiotic resistance. Critics point out that while the immediate benefits of reducing SSIs are clear, the long-term consequences of widespread antibiotic use may be detrimental. The World Health Organization (WHO) and other health authorities have cautioned against the indiscriminate use of antibiotics due to their potential to foster resistant bacterial strains^[2]^. This perspective emphasizes a need for a more nuanced approach to antibiotic prophylaxis, advocating for judicious use based on individual patient risk factors rather than blanket policies.

The literature reflects this tension; while many studies support prophylactic use in specific contexts, others suggest that antibiotics may be unnecessary in certain clean surgeries where infection rates are low^[3]^. Critics argue that guidelines should evolve to reflect these findings, advocating for more tailored approaches that consider patient-specific factors such as underlying health conditions and the nature of the surgical procedure.

The debate extends into clinical guidelines as well. Despite recommendations from bodies like the US Centers for Disease Control and Prevention (CDC) and the National Institute for Health and Care Excellence (NICE) against prolonged postoperative antibiotic use, many clinicians continue to prescribe them as a precautionary measure. This discrepancy highlights a gap between evidence-based recommendations and clinical practice. Some practitioners feel that the potential risks of SSIs justify the continued use of prophylactic antibiotics, especially in procedures where infection risk is perceived to be higher^[4]^.

Furthermore, there is an argument to be made about the evolving nature of surgical techniques and patient demographics. As surgical methods improve and patients become increasingly complex – often with comorbidities that elevate their risk profiles – the rationale for using prophylactic antibiotics may also change. Proponents of this view argue for ongoing research to refine guidelines based on emerging evidence. Ethical considerations also play a significant role in this discourse. The decision to administer prophylactic antibiotics involves weighing the benefits against potential harms, including allergic reactions or gastrointestinal disturbances experienced by some patients. Additionally, there is an ethical obligation to consider public health implications; excessive antibiotic use contributes to individual patient risks and broader societal challenges related to antimicrobial resistance. As such, some experts advocate for a more collaborative approach involving multidisciplinary teams that include surgeons, infectious disease specialists, and pharmacists when developing protocols for antibiotic use in surgical settings^[5]^. This collaboration could lead to more informed decisions that balance patient needs with public health considerations.

The literature surrounding antibiotic prophylaxis in SSIs presents a complex interplay of evidence supporting its efficacy alongside significant concerns regarding resistance and ethical implications. While Tang *et al.*’s systematic review strongly supports the continued use of prophylactic antibiotics in specific surgical contexts, it highlights the need for ongoing dialogue within the medical community about best practices. As new evidence emerges and surgical techniques evolve, it will be crucial for clinicians to adapt their approaches based on a comprehensive understanding of both immediate patient outcomes and long-term public health implications. Fostering an environment where evidence-based practice coexists with ethical responsibility will be critical in navigating this contentious yet vital aspect of surgical care.

## Data Availability

None.
